# Impact of Different *Helicobacter pylori* Eradication Therapies on Gastrointestinal Symptoms

**DOI:** 10.3390/medicina57080803

**Published:** 2021-08-05

**Authors:** Antonio Mestrovic, Josko Bozic, Katarina Vukojevic, Ante Tonkic

**Affiliations:** 1 Department of Gastroenterology, University Hospital of Split, 21000 Split, Croatia; amestrovic@kbsplit.hr (A.M.); ante.tonkic@mefst.hr (A.T.); 2 Department of Pathophysiology, School of Medicine, University of Split, 21000 Split, Croatia; 3 Department of Anatomy, School of Medicine, University of Split, 21000 Split, Croatia; katarina.vukojevic@mefst.hr; 4 Department of Internal Medicine, School of Medicine, University of Split, 21000 Split, Croatia

**Keywords:** *Helicobacter pylori*, eradication therapy, quality of life, Gastrointestinal Symptom Rating Scale, hybrid therapy, concomitant therapy

## Abstract

*Background and Objectives*: *Helicobacter pylori (H. pylori)* infection impairs quality of life. However, whether eradication therapy ameliorates gastrointestinal symptoms remains questionable. The main objective of this study was to evaluate the influence of *H. pylori* eradication therapy on gastrointestinal symptoms. *Materials and Methods*: A total of 140 patients, 59 women and 81 men, with a mean age of 61 and suffering from *H. pylori* infection in the University Hospital of Split, Croatia, were enrolled in the study. Patients were randomly assigned to either concomitant or hybrid therapies. The Gastrointestinal Symptom Rating Scale (GSRS) questionnaire was completed by patients prior to and after the eradication therapy. *Results*: In both groups, the total GSRS score improved significantly after therapy. In the concomitant group, the abdominal pain score, reflux symptoms score and indigestion score decreased significantly after therapy. In the group with hybrid therapy, all five groups of symptoms (abdominal pain, reflux symptoms, indigestion, diarrhea and constipation) significantly decreased after therapy. Patients with adverse events had significantly higher total GSRS scores after eradication therapy. *Conclusions*: *H. pylori* eradication therapy could alleviate gastrointestinal symptoms regardless of the treatment used, but the favorable effect seemed to be more pronounced after hybrid therapy.

## 1. Introduction

*Helicobacter pylori (H. pylori)* infection still represents a public health burden. *H. pylori* remains a crucial element in the development of conditions such as gastritis, gastric and duodenal ulcers, gastric adenocarcinoma and mucosa-associated lymphoid tissue lymphoma (MALT) [1,2]. Furthermore, *H. pylori* is a well-known factor in the carcinogenesis of stomach cancer [3]. Although the presence of *H. pylori* virtually always leads to chronic gastritis, it is asymptomatic in over 80% of cases [4]. However, epigastric pain and dyspeptic symptoms can be caused by *H. pylori* infection, and it might impair various aspects of a patient’s quality of life (QOL). Current guidelines require the treatment of *Helicobacter pylori* infection regardless of symptomatology [2,5]. Although *H. pylori* infection has been a subject of numerous studies for decades, the number of studies involving the impact on the quality of life in patients with *H. pylori* is limited. A causal link between dyspeptic symptoms and *H. pylori* infection has been suggested mainly through studies of infected patients with uninvestigated or functional dyspepsia. A systematic review of 21 randomized clinical trials concluded that eradication therapy had a small but statistically significant effect in *H. pylori*-positive non-ulcer dyspepsia (estimated number needed to treat = 14) [6]. A randomized, single-blind, placebo-controlled study on *H. pylori* eradication for functional dyspepsia concluded that efficacy of eradication had symptom-based tendencies in patients with functional dyspepsia (FD) and that it might be effective in the subgroup of FD patients with epigastric pain syndrome [7]. On the other hand, there is even less interest in the investigation of quality of life in the context of eradication therapy. Few studies have shown that the QOL of patients with epigastric symptoms improved after the eradication of *H. pylori* [8,9].

One of the recognized tools for the assessment of gastrointestinal symptoms is the Gastrointestinal Symptom Rating Scale (GSRS) questionnaire. Although it has been frequently used for the assessment of symptomatology in peptic ulcer disease, reflux disease and functional dyspepsia, its use for *H. pylori* infection remains scarce [8,10,11,12].

As data regarding the comparison of adverse effects between concomitant and hybrid therapies are insufficient, the aim of this study was to determine the influence of different eradication protocols on gastrointestinal symptoms in patients with *H. pylori* infection.

## 2. Materials and Methods

### 2.1. Design Overview

#### 2.1.1. Patients

We conducted a prospective study based on the previous randomized controlled trial comparing concomitant and hybrid therapy [13]. From July 2018 to August 2019, patients who visited the University Hospital of Split, Croatia and who were *H. pylori* positive were included in the study. In this previous study, a total of 140 patients (59 women and 81 men, with mean age of 61) with proven *H. pylori* infection were enrolled [13]. Exclusion criteria were age less than 18 years; the previously unsuccessful application of empirical *H. pylori* eradication therapy; malignant disease of the stomach or any other site; taking proton pump inhibitors (PPI), H2 antagonists, bismuths or antibiotics (amoxicillin, metronidazole, clarithromycin) during the last month; associated comorbidity (renal insufficiency, mental illness); history of allergies to proton pump inhibitors or antibiotics (amoxicillin, metronidazole, clarithromycin); pregnancy and lactation; or refusal to participate in the survey [13]. Patients were randomly assigned using a computer generating sequence to either concomitant (esomeprazole 40 mg, amoxicillin 1 g, metronidazole 500 mg, clarithromycin 500 mg twice daily for 14 days) or hybrid therapy (esomeprazole 40 mg and amoxicillin 1 g twice daily for 14 days with added metronidazole 500 mg and clarithromycin 500 mg twice daily in the last 7 days) [13]. Upon a baseline examination before therapy, the patients were given the GRSR questionnaire. One month after the end of therapy, while blinded to the results of the previous questionnaire survey and the results of the *H. pylori* eradication therapy, they were again given the GSRS questionnaire.

#### 2.1.2. Gastrointestinal Symptom Rating Scale Questionnaire

The Gastrointestinal Symptom Rating Scale (GSRS) is a specific, 15-item questionnaire for patients with gastrointestinal symptoms. The questionnaire has a seven-point graded Likert-type scale, where 1 represents absence of symptoms and 7 represents very troublesome symptoms. The reliability and validity of the GSRS are well-documented, and norm values for a general population are available [10,11,12,14,15,16]. In this study, we used the Croatian version of the questionnaire, which was previously translated from the original, English version. Subsequently, we translated the Croatian version back to English in order to avoid possible errors in translation which could lead to misinterpretation. The sum of the scores for all 15 items was regarded as the GSRS total score. The scores for the five symptom categories, namely, the reflux score, abdominal pain score, indigestion score, diarrhea score and constipation score, were obtained by calculating the means of the scores of the five items describing each symptom pattern. The higher the overall score, the more severe the symptoms. Dyspepsia could be defined through the following two questions in the GSRS questionnaire: “Have you had abdominal pain located in the upper abdomen for at least one week”, and “Have you ever had heartburn or acid regurgitation almost daily for at least one week”. A positive answer to at least one of the questions defined dyspepsia, as used by Asfeldt et al. [17].

All participants provided written informed consent. The study was performed in accordance with the principles of good clinical practice from the Declaration of Helsinki, approved by the Ethics Committees of the University Hospital of Split (as from April 2018; approval number 500-03/18-01/13) and the University of Split School of Medicine (as from April 2018; approval number: 003-08/18-03/0001) and registered as a clinical trial (Clinical Trials, gov: NCT03572777).

### 2.2. Statistical Analysis

The statistical software SPSS version 25 for Windows (IBM Corp, Armonk, NY, USA) was used for statistical data analysis. Data were expressed as medians and interquartile ranges. The Mann-Whitney U test was used for independent samples, and the Wilcoxon test was used for paired samples. The Kolmogorov-Smirnov test was used to test the normality of the data. The statistical significance was defined as *p* < 0.05.

## 3. Results

A total of 140 patients were randomly assigned to concomitant (*n* = 69) and hybrid (*n* = 71) groups. In the previous study, there was no significant difference between groups regarding eradication rates (84.1% (58/69) and 83.1% (59/71), respectively) in the intention-to-treat analysis (*p* = 0.878) and 96.7% (58/60) and 95.2% (59/62) in the per-protocol analysis (*p* = 0.675) [13]. However, adverse events, which were thoroughly discussed in our previous study, were significantly higher in the concomitant than in the hybrid group (33.3% vs. 18.3%, *p* = 0.043) [13].

In the concomitant group, the total GSRS result was significantly lower after than prior to eradication therapy (median 23 (IQR 18-28) vs. median 19 (15.75–24); *p* < 0.001), as shown in Table 1. Abdominal pain, reflux symptoms and maldigestion symptoms scores were significantly lower after than before eradication therapy (Table 1). There were no significant differences in diarrhea and constipation symptom scores before and after eradication therapy (Table 1).

In the hybrid group, the total GSRS result was significantly lower after in comparison to before eradication therapy (median 28 (IQR 20–39.5) vs. 22 (IQR 18–31); *p* < 0.001), as shown in Table 2. In all five symptom groups, scores were significantly lower after than before eradication therapy (Table 2).

When we compared changes in GSRS scores in the concomitant and hybrid groups after therapy in relation to the basal data, there was no significant difference among groups (median 2 (IQR 0–5) vs. median 2 (IQR 0–6.75); *p* = 0.530).

Patients with adverse events during eradication therapy had significantly higher total GSRS scores after than prior to eradication therapy: median 22 (IQR 18–31) vs. median 19.5 (IQR 16–25); *p* = 0.041). However, comparing changes in GSRS scores prior to and after therapy in relation to the basal data, there was no significant difference between patients who had adverse events and those who didn’t have adverse events (median 2.5 (IQR 0–8) vs. median 2 (IQR 0–5); *p* = 0.870).

## 4. Discussion

*H. pylori* infection, commonly associated with dyspepsia, impairs quality of life [9,18]. However, the possible impact of *H. pylori* infection on gastrointestinal symptoms has been rarely investigated thus far. To date, even less interest has been shown in terms of the possible impact of eradication therapy itself on quality of life and various gastrointestinal symptoms. The intention of this study was to highlight the influence of two different eradication regimes on quality of life in the aspect of gastrointestinal symptoms through a validated questionnaire. Our results indicated the beneficial effect of eradication therapy on quality of life in patients with gastrointestinal symptoms due to *H. pylori* infection. Both concomitant and hybrid eradication therapies had beneficial impacts on gastrointestinal symptoms in their total GSRS scores. A similar positive effect of eradication therapy has also been observed in earlier studies [18,19]. Additionally, a multicenter prospective study performed in 15 referral institutions on 165 *H. pylori* infected patients demonstrated that quality of life could be improved regardless of eradication outcome [18]. In the same study, a lower quality of life score prior to eradication therapy was associated with an improved quality of life after therapy [18]. Our results also suggest that quality of life can be improved regardless of the choice of eradication therapy because we used two therapy protocols that yielded similar success in the treatment of *H. pylori* infection in the previous study, with a greater than 95% rate of eradication in per-protocol analysis [13].

Focusing on individual symptoms, in the concomitant group, a positive effect was demonstrated in the cases of reflux symptoms, abdominal pain and maldigestion. However, in the group with hybrid therapy, a beneficial effect was additionally shown in the case of diarrhea and constipation symptoms. The main difference between concomitant and hybrid therapy is the 7-day shorter administration of clarithromycin and metronidazole, which can result in less adverse events [13]. As expected, patients who had more adverse events also had a poorer overall GSRS score with no statistically significant difference after eradication therapy. Reflux symptoms, maldigestion and abdominal pain are frequently associated with symptomatic *H. pylori* infection. Yet, the only beneficial role of successful eradication therapy, in the case of dyspeptic symptoms, is well-known, as previous studies have shown [8,19]. *H. pylori* gastritis can be the cause of dyspepsia in a subset of patients [20,21]. Systematic reviews showed that *H. pylori* eradication therapy was beneficial for the improvement of upper gastrointestinal symptoms in functional dyspepsia [6,22]. On the other hand, a large cross-sectional analysis of 3005 Japanese subjects concluded that *H. pylori* infection and history of eradication did not affect acid-related dyspepsia symptoms [23].

The impact of eradication therapy on reflux symptoms still remains controversial [24]. On the other hand, one study suggested that reflux symptoms might be improved one year after eradication [9]. According to our results, both eradication therapies alleviated the mentioned symptoms, regardless of the choice of therapy.

The hybrid therapy group showed better outcomes in GSRS scores after eradication in terms of constipation and diarrhea. The link between *H. pylori* infection and diarrheal syndrome is controversial. An assumption is that hypochlorhydria as a consequence of chronic gastritis increases the risk of diarrhea [25]. Diarrhea is also often regarded as a common adverse event of eradication therapy, which complicates symptom scoring. This study concluded that in the hybrid therapy group, diarrheal symptoms were less present after eradication therapy. Until now, only a few studies have shown less frequent diarrhea after therapy, but it has been combined with probiotic use [26,27]. Additionally, eradication therapy might improve constipation symptoms, as shown by Murata et al. [28]. In the case of constipation and diarrhea, we can assume that less antibiotics may have a less negative influence on gut microbiota, which can lead to less disturbance in bowel movement.

*H. pylori* has been considered a possible factor influencing the gut microbiota [26,29,30,31]. Furthermore, *H. pylori* infection can contribute to gastric microbial dysbiosis, which may be involved in carcinogenesis [31]. However, Guo et al. showed that successful *H. pylori* eradication potentially restored gastric microbiota to a similar status as found in uninfected individuals and showed beneficial effects on gut microbiota [31]. On the other hand, Chen et al. conducted a randomized clinical trial which defined disturbance in the gut microenvironment after eradication [26].

Our study has several notable limitations. In this study, we evaluated GSRS scores one month after eradication therapy. For some symptoms such as reflux syndrome, we can expect improvement even one year after eradication therapy, as Hirata et al. demonstrated [9]. Meanwhile, patients with *H. pylori*-associated dyspepsia, by definition, should obtain sustained symptomatic relief for six months after therapy according to the Kyoto definition [5]. However, our results showed improvement in both groups even after one month. It is possible that a longer observation time is necessary to ensure that these improvements are permanent. We also need to point out that we did not compare hybrid and concomitant groups regarding GSRS scores. The reason was the differences in baseline findings in the GSRS scores among groups before therapy. Thirdly, the study of the gut microbiota itself before and after eradication therapy would be prudent and would certainly put forward more accurate insight into microenvironmental changes. Studies with other standard eradication protocols should be performed to draw conclusions on the overall influence on gastrointestinal symptoms. Finally, as different types of H. pylori strains might induce different symptoms or alter their severity, and as we did not perform an analysis of H. pylori strains, it is possible that the observed results are, in part, a consequence of the different strains of *H. pylori* involved [32].

## 5. Conclusions

Both concomitant and hybrid eradication of *H. pylori* therapy improved quality of life, even one month after therapy. Both concomitant and hybrid therapy had a beneficial role in reflux symptoms, maldigestion and abdominal pain. However, hybrid therapy improved constipation and diarrhea as well, possibly because of less antibiotic usage. Nonetheless, further studies are warranted in order to confirm these inferences.

## Figures and Tables

**Table 1:** Results of GSRS questionnaire before and after therapy in concomitant group.

Parameter	Baseline (*n* = 69)	After Therapy (*n* = 69)	*p* ^a^
Abdominal pain	5 (3–6.25)	3 (3–5)	<0.001
Reflux symptoms	3 (2–4)	2 (2–4)	0.015
Maldigestion	6 (5–9)	5 (4–7)	0.001
Constipation	3 (3–4)	3 (3–4)	0.328
Diarrhea	3 (3–4)	3 (3–3)	0.146
Total	23 (18–28)	19 (15.75–24)	<0.001

Data are presented as median and interquartile range. ^a^ Wilcoxon test. GSRS: Gastrointestinal Symptom Rating Scale.

**Table 2:** Results of GSRS questionnaire before and after therapy in hybrid group.

Parameter	Baseline (*n* = 71)	After Therapy (*n* = 71)	*p* ^a^
Abdominal pain	5 (3–7.75)	4 (3–6)	<0.001
Reflux symptoms	4 (2–6)	2 (2–4)	<0.001
Maldigestion	9 (6–14)	7 (4–9)	<0.001
Constipation	5 (4–6)	4 (3–6)	0.002
Diarrhoea	3 (3–5)	3 (3–4)	0.003
Total	28 (20–39.5)	22 (18–31)	<0.001

Data are presented as median and interquartile range. ^a^ Wilcoxon test.

## Data Availability

All data is available from the corresponding author.
